# HIV and sexually transmitted infections: responding to the “newest normal”

**DOI:** 10.1002/jia2.25164

**Published:** 2018-07-10

**Authors:** Kenneth H Mayer, Henry de Vries

**Affiliations:** ^1^ Fenway Health The Fenway Institute Boston MA USA; ^2^ Department of Infectious Diseases Beth Israel Deaconess Medical Center Boston MA USA; ^3^ Harvard Medical School Harvard University Boston MA USA; ^4^ Department of Infectious Diseases Public Health Service of Amsterdam Amsterdam The Netherlands; ^5^ Department of Dermatology Academic Medical Centre University of Amsterdam Amsterdam The Netherlands; ^6^ Amsterdam Infection and Immunity Institute (AI&II) Academic Medical Centre University of Amsterdam Amsterdam The Netherlands

**Keywords:** STD, STI, PrEP, HIV care continuum, Treatment

Although the first case reports of AIDS were in men who had sex with men (MSM) 1. and HIV is most commonly spread heteroxexually 2, the epidemic has usually been addressed differently than other sexually transmitted infections (STI). Initially, this was because it emerged as a lethal, untreatable, rapidly spreading epidemic. Focused AIDS research, palliative patient care programs, and community activism evolved to deal with the mounting morbidity, and to speed the development of highly active antiretroviral therapy (HAART). In contrast, “classical” STI care and research were already embedded in well‐established settings. Thus, from the beginning, HIV and STI were often addressed through separate programs.

However, subsequent research demonstrated STI and HIV synergism 3. The co‐occurence of STI, particularly genital herpes, was shown to facilite the spread of HIV 4. Because of the effects of STI in potentiating HIV acquisition and transmission, several randomized controlled trials were undertaken to determine whether STI treatment and/or prophylaxis could decrease HIV incidence 5, 6, 7. Only the Mwanza trial that treated symptomatic STI demonstrated a modest impact of STI treatment in HIV incidence 5. The other studies did not find that mass treatment and/or syndromic management were sufficient to arrest HIV spread. STI management as a primary HIV prevention strategy was superseded once safe and effective HAART became widely accessible, since it was shown that suppressive treatment significantly decreased HIV transmission 8, 9.

The proof that HAART could decrease HIV transmission 10, 11, and that pre‐exposure prophylaxis (PrEP) could decrease HIV acquisition 12, uncoupled epidemiologic synergy between STI and HIV 13. HIV‐infected individuals who adhered to their medication, whose virus was suppressed, would not transmit HIV to partners 14, and PrEP adherence provided substantial protection against HIV acquisition for high risk uninfected people 15. Although condoms remain highly effective in preventing STI transmission 16, their decreased use in this era of antiretroviral optimism has been associated with bacterial STI increasing globally. At present, there are more than 1 million new curable STI occurring daily globally 17, and although HIV incidence is slowly declining in several settings, there still are close to 2 million new HIV infections annually, and nearly 40 million people living with the virus 18.

The “new normal” of HIV and STI spread becoming unlinked offers unique opportunities to control both epidemics 19, particularly in the light of increasing sophistication in understanding mucosal biology, as well as the behavioural and sociological factors potentiating HIV and STI spread 20. At the 22nd International AIDS Conference in Amsterdam in July 2018, for the first time, there will be a two‐day pre‐conference focusing exclusively on HIV and STIs: “STI 2018: Understanding and Addressing the HIV and STI Syndemics.” This meeting is designed to review the contemporary epidemiology of HIV and STIs in key populations, as well as in generalized HIV epidemic settings. The presentations will focus on the contemporary milieus in which HIV and STI spread, concentrating on specific groups, such as sex workers, MSM, and young women, and newer contexts, for example internet‐based sexual “cruising” sites, and the use of “designer drugs” to enhance sexual experiences (“chemsex”) 21. Presenters will discuss new insights regarding how different mucosal microbiomes may affect HIV‐STI spread, including the mechanisms of how STI‐mediated chronic inflammation potentiates HIV transmission and susceptibility. As new prevention technologies are developed, one recent example of the need to focus on the mucosa was the finding that bacterial vaginosis attenuated the effectiveness of topical tenofovir gel to prevent HIV transmission 22.

Presenters at STI 2018 will discuss how the advent of innovative technologies, including self‐ and point‐of‐care HIV and STI testing, and new service delivery models (e.g. express clinics using computer interfaces), home sample collection, rapid nucleic amplification testing, and SMS texting to deliver test results in an expedited manner) to more effectively deliver clinical services 23. Other forward‐looking approaches will be discussed, for example the use of the Internet for partner notification 24, and the role of sexual health apps to engage high risk individuals, particularly youth, in accessing screening and preventive services. The conference will bring together community, clinical, and public health leaders to address some of the unintended consequences emerging from the creation of comprehensive sexual health promotion programs, including stock outs, supply chain issues, the need to engage industry to develop new medicines, given the emergence of multi‐drug resistant STI organisms and identification of new STI pathogens 25, all while minimizing stigma and ensuring human rights. An example of the multidisciplinary approach of the meeting will be a discussion of the opportunities and challenges in expanding the use of nucleic amplification testing to detect “hard to culture” pathogens. Wider testing offers the promise of improved surveillance and more selective use of antibiotics, particularly important, given the spectre of multi‐drug resistant STI pathogens, for example gonorrhoea. But, the newer tests and novel antimicrobials will be expensive, hence the need for innovative public health approaches to increase access. Another much debated issue, syndromic management of STI will be reviewed. It is controversial 26, since the majority of chlamydia and gonorrhoea infections in women are asymptomatic and would not be captured through syndromic management alone, resulting in untreated genital infections 27. The meeting will discuss newer diagnostics and improved service delivery models, which offer the promise of enhanced epidemic control, but will require political will to support the added costs of innovations and implementation of best practices.

Integration of HIV and STI control requires a nuanced understanding of the salient cultural and behavioural factors potentiating HIV susceptibility and transmission. Individuals at greatest risk for HIV and STI are often members of socially marginalized populations, whose lived experiences and internalized stigma may result in high rates of concomitant depression, substance abuse, and decreased self‐efficacy 28, 29, often resulting in avoidance of healthcare settings, where discrimination may be anticipated and experienced 30, 31, 32, 33. The high co‐prevalence of HIV, STI, and socio‐behavioural challenges in vulnerable populations function as syndemics, which require integrated and multifaceted approaches to engage those at greatest risk for HIV and STI in programs to increase testing, linkage to treatment and preventive services. Frequently concomitant societal rejection may result in avoidant health seeking behaviour, delaying diagnoses and ineffective partner notification, impeding public health control of STI and HIV epidemics. Healthcare providers need to be taught about the provision of culturally competent care, so that vulnerable populations seek clinical services, leading to earlier diagnoses and the prevention of further HIV and/or STI spread. The support of knowledgeable healthcare workers is particularly critical in societies with traditional social norms, that circumscribe the sexual autonomy of women and criminalize same sex behaviour. Fortunately, an increasing array of in‐person and on‐line resources (e.g. http://www.lgbthealtheducation.org) are available to train clinicians in the provision of culturally competent care for sexual and gender minority people.

The increasing ability to control the HIV epidemic through the use of HAART can guarantee HIV‐infected people long and healthy lives, and PrEP means that at risk persons do not need to become infected. These advances are welcome developments. But given that sexual expression is part of being human, in an era when HIV is more controllable, increases in the global STI burden are not surprising. The challenge for researchers, clinicians, and public health officials is to understand how to best promote sexual health in this new age. The desirable benefits of improvements in HIV treatment, diagnostic capabilities for HIV and STI, and educational digital media create new challenges and opportunities for key stakeholders and civil society to limit STI spread while respecting individual decisions about sexual expression. The HIV‐STI pre‐conference in Amsterdam is intended to expand understanding that mantras like “Getting to Zero” (zero new HIV infections) will never be achieved without addressing the potentiating role of STI in the global HIV pandemic, in addition to responding to other drivers of HIV spread, including economic and gender inequality, and other human rights challenges. Creative approaches to the integration of HIV and STI research and programs should allow for more efficient use of resources to decrease STI‐associated morbidity and to improve global sexual health.

## Competing interests

None.

## Authors’ contributions

KHM and HV have contributed to the preparation of the manuscript, read and approved the final draft.
